# Development and evaluation of robotic detection technology for assessing autism

**DOI:** 10.3389/fpsyt.2025.1636560

**Published:** 2025-09-26

**Authors:** Wing-Chee So, Elsa Wong, Wingo Ng, Sally Lay, Wynes Wong, Ming-Ting So, Xiao-Han Lee, Yuen-Yung Lee

**Affiliations:** ^1^ Department of Educational Psychology, The Chinese University of Hong Kong, Hong Kong, Hong Kong SAR, China; ^2^ NEC Hong Kong Limited, Hong Kong, Hong Kong SAR, China

**Keywords:** autism, assessment, autonomous robot, detection, children

## Abstract

**Objective:**

An objective and standardized assessment for assessing autism is needed. This study aimed to develop and validate robotic detection technology for assessing autism. The robot HUMANE, installed with computer vision and linked with face and motion recognition technology, autonomously detected atypical eye gaze and repetitive motor movements, two of the features of autism, while narrating stories. It autonomously prompted the child if they did not establish eye gaze with the robot or produced motor movements for five seconds continuously.

**Method:**

The study involved 119 children aged between three and six years old (M=4.53, SD=1.89; 38 females) and included children confirmed or not confirmed with autism. They all received the Autism Diagnostic Observation Schedule—second edition (ADOS‑2), the standard diagnostic tool for autism. HUMANE’s detection performance – the number of robot prompts and the cumulative duration of inattentiveness/improper posture – was then evaluated against the calibrated severity score of ADOS-2.

**Results:**

Our results showed that the average sensitivity and specificity of the detection reached 0.80, the Diagnostic Odds Ratio was beyond 30, and the AUC was .85.

**Discussion:**

These results indicate that the robotic detection technology of atypical eye gaze and repetitive motor movements can contribute to the diagnostic process to identify the presence or absence of autism.

## 1 Introduction

Autism Spectrum Disorder (hereafter, autism) is a complex neurological disorder. In the U.S. approximately one in 31 children aged eight years has been diagnosed as being on the autism spectrum (1). Early diagnosis is paramount for early intervention and is also critical for successful inclusion of autistic individuals into society. Clinicians diagnose autism based on criteria from the Diagnostic and Statistical Manual of Mental Disorders - fifth edition (DSM-5; 2), children’s behavior and social skills elicited in the Autism Diagnostic Observation Schedule - second edition (ADOS-2; 3), and/or interviews with the parents in the Autism Diagnostic Interview - revised (ADI-R; 4). While significant training is required for clinicians to become proficient in diagnosing autism, it is inevitable that their decisions will be based on subjective judgement. Different diagnoses can be made by different clinicians (5). Given the heterogeneity of autism, only 60% to 70% of autism diagnoses made by licensed and experienced clinicians are made with certainty (6, 7). Furthermore, there is limited availability of experienced clinicians (8), often resulting in long waiting times.

Given the challenges of human-based diagnosis, there is a pioneering development of data-driven information and communication technology (ICT) solutions including software applications, wearable devices, robotics, augmented/virtual reality help to screen or assess autism earlier than the current average age of diagnosis (9, 10). Such techniques can improve objectivity and quantify the diagnostic process, assessment, and evaluation of learning outcomes. Moreover, they can automatically and accurately detect impaired social interactions and repetitive and stereotyped behavior that are the key features of autism. The present study focused on the development of robotic technology in assessing autism. Specifically, it aimed to validate a robotic screening tool that detects the hallmark autism symptoms of atypical eye movement patterns in autistic children (11) and repetitive motor manners.

According to the empathizing-systemizing theory (12), robots are operated on predictable and lawful systems, creating a favorable learning environment for autistic children who may struggle to learn in an unpredictable and distracting environment. Social robots have been widely used in therapy for autistic people in recent decades (see reviews in 13–15). As early as 2005, Scassellati had proposed the idea of using social robots to address critical issues in autism diagnosis. However, in comparison to empirical research investigating the effectiveness of robot-based intervention, there has been little examination as to whether social robots can be used for screening and assessing or diagnosing autism (see reviews in 16).

A few studies have explored the supporting role of robots in screening autism (11, 17–22), largely focusing on the detection of one autism feature. Most of these studies adopted either the Wizard-of-Oz (WoZ) paradigm or the semi-autonomous strategy. Under the WoZ paradigm, the human experimenter commands the robot for actions to be completed. In Arent et al et al, (17), a NAO robot was remotely controlled by an experimenter using the standard NAO GUI when engaging with children in interactive dyadic games (“Dance with me” and “Touch with me”). Children’s turn-taking behaviors were then rated by human researchers. Their findings showed that autistic children presented a deficient level of turn-taking behavior, compared with the typically developing children. However, this result was based on human ratings that might lack objectivity.

Also adopting the WoZ paradigm, a pilot study by Del Coco et al. (18) had the therapist command the robot, Zeno, through a tablet when engaging the child. This differed from Arent et al.’s study in that the robot was installed with the tablet camera that processed the videos of the child and generated multiple behavioral cues. Those behavioral data were then objectively analyzed by algorithms for automatic detection and computation of eye gaze, head pose, and facial expression. This system could detect autism features in the most severely autistic child.

In a different approach from the aforementioned studies, Ramírez-Duque and his team adopted a semi-autonomous paradigm and designed a robot-assisted framework, where the robot, ONO, interacted with the child and modified their behavior (e.g., direction of eye gaze, facial expression, and response to rewards) based on an algorithm (22). ONO was equipped with a sensor that detected the child’s nonverbal behaviors, including looking toward an object, toward the robot, and toward the therapist, and pointing to or responding to a prompt from the therapist. These behaviors were analyzed by a pipeline algorithm implemented in the machine-learning neural models. The findings showed that children at risk of an autism diagnosis tended to be more interested in interacting with and looking at the robot than those without risk. However, only six children, three typically developing and three autistics, were involved. Given the small sample size, it is difficult to draw implications from this that the robot detection system can screen for autism.

In a recent exploratory cross-sectional case control study, So and colleagues programmed the robot HUMANE, installed with computer vision and linked with recognition technology, to screen autism based on its detection of children’s direction of eye gaze toward the robot (11). In their study, the Autism Care Windows application regulated HUMANE’s activities, and upon the initiation of gaze detection, the program extracted images from HUMANE at a rate of 8 frames per second and consistently transmitted them to NeoFace^®^, which operated in the cloud using a 5G mobile network. The technology analyzed the incoming frames, stored the gaze findings in a database for report generation, and transmitted metadata to HUMANE for vocalization. Upon acquiring the metadata, HUMANE deciphered each communication and evaluated subsequent actions. Should the child sustain their eyes on HUMANE, the robot continued in narrating the story. Nevertheless, the robot’s narration would cease and HUMANE would prompt the child to refocus on it if the child surpassed the 5-second threshold of not concentrating on it. Upon the child’s gaze returning to HUMANE, the off-focus timer was reset, and HUMANE commended the child before continuing the narration. Children aged between three to eight (N = 199) participated in the study. After receiving instruction from the human experimenter, HUMANE narrated a story to a child and autonomously prompted them to return their eye gaze to the robot if they looked away from the robot and praised them when eye gaze was quickly re-established after a prompt. Its detection of eye gaze toward the robot reached a reliability of 0.90. Additionally, using the pre-specified reference standard, Autism Spectrum Quotient-10 items (Hong Kong Chinese Child Version)–(AQ-10-Child-HK); 23), the sensitivity and specificity of using the number of prompts made by the robot and the duration of inattentiveness detected by the robot to discriminate autism from non-autism reached 0.88 and 0.96 respectively, and the Diagnostic Odds Ratios were 191.18 and 434.48 respectively. These results indicate that social robots can screen autism based on the robot’s detection of atypical eye patterns.

However, So et al (11) study used the pre-specified cut-off of a parental self-report, AQ-10-Child-HK, that is considered as a screening tool, but does not reach the diagnostic standard. To validate the robotic screening tool, it is necessary to use the standard autism assessment method adopted by clinicians, the Autism Diagnostic Observation Schedule - second edition (ADOS-2; 3). Another limitation is that the robotic screening tool invented in So et al.’s study focuses solely on atypical eye gaze, which is only one of the impairments presenting in autistic children in social communication and interaction. This limits its clinical applicability as it is difficult or impossible to determine if a child is on the autism spectrum based on one feature only (18).

Our study aimed to address these limitations by first adopting the calibrated severity scores of ADOS-2 (3), considered to be the “gold standard” in the assessment of autism, as the reference standard. Second, we programed the same autonomous social robot to detect more than one autism feature when engaging with children. In addition to recognizing lack of eye gaze in social interaction and communication, the system also detected specific kinds of restrictive and repetitive behavior, another diagnostic criterion listed in the Diagnostic and Statistical Manual of Mental Disorders (DSM-5; 2). Since restrictive and repetitive behavior includes a broad category of behaviors such as preoccupation with restricted patterns of interest, adherence to specific, nonfunctional routines, repetitive motor manners, and preoccupation with parts of objects, it is difficult for the system to detect all these behaviors. Among different kinds of restrictive and repetitive behaviors, this study focused on repetitive motor manners, specifically hand mannerisms (e.g., hand flapping/waving) and complex mannerisms (e.g., body rocking, pacing back and forth, spinning circles) that could be manifested in improper sitting postures. It is common that autistic children struggle to sit still, possibly due to sensory-motor issues. Their problems in processing sensory information are in turn translated into repetitive motor movements.

Repetitive movements are not unique to autistic children but are also present in non-autistic children, such as those with intellectual disabilities and language disorders (e.g., 24). However, young children who are later diagnosed with autism are perceived by their caregivers as having more prevalent and severe repetitive movements than those who are not diagnosed with autism (25, 26). The scores of repetitive and ritualistic behaviors in ADOS-2 also significantly enhanced the predictive power in classifying young children with autism, developmental delays, and typical development years later (27).

To sum up, this study will advance the development of robotic screening tools by detecting two features: atypical eye gaze and repetitive motor movements. Our robotic detection system processes these two features independently based on their corresponding rules of recognition and scoring algorithms. Individual indexes for each feature and composite indexes across both features were generated to facilitate a comprehensive and objective screening process. For the detection of atypical eye gaze, we followed the individual indexes generated in So et al (11) study: the number of times the robot prompted the child when not establishing eye contact with the robot for a certain period and the cumulative duration of inattentiveness (in seconds). For the detection of repetitive motor manners we generated similar individual indexes, of how often the robot prompted the child when not sitting properly for a certain period and the cumulative duration of improper sitting posture (in seconds). The composite indexes were the total number of prompts made by the robot when detecting atypical eye gaze and improper sitting posture as well as the total duration of inattentiveness and improper sitting posture. The indexes, at individual and composite levels, were then evaluated using ADOS-2 as the reference standard. We hypothesized that the composite indexes would identify the presence or absence of autism, with the sensitivity and specificity reaching at least 0.8 and the Area under the Curve reaching at least 0.7. We also hypothesized that the composite indexes would have a greater discriminative power than the individual indexes.

## 2 Methods

### 2.1 Participants

A total of 119 children participated, aged between three and six years (M = 4.53, SD = 1.89; 38 females). We recruited the sample from this age range because most children suspected of being autistic are referred to pediatricians or psychologists at assessment centers at around age four in Hong Kong. The study aimed to validate a screening tool for autism using a social robot to identify autistic children at a younger age than is currently the case, hence we recruited children aged three to six. **Table 1** shows the demographics of the participating children, 36.13% of whom had been diagnosed with autism, 29.41% of whom were suspected to have autism, and the remainder of whom (34.46%) were not thought by their parents to have autism. Participants were recruited from autism treatment centers, kindergartens, and primary schools in Hong Kong. Children with known vision or hearing deficits and those who did not know Cantonese (Chinese), were excluded. All parents or legal guardians of the participants gave written informed consent, and the study protocol was approved by the Survey and Behavioral Research Ethics (SBRE) Institutional Review Board at the first author’s institution.

**Table 1:** Demographic statistics of the participants in this study.

Variables	Confirmed diagnosis of autism	Suspected to have autism	No concerns of having autism	Total
N	43	35	41	119
Mean age in months (SD)	55.28 (12.87)	51.36 (16.54)	53.29 (14.21)	--
Range in months	40-68	36-70	38-69	--
N of males	36	32	22	90
ADOS-2 CSS Mean SD Range	7.66 1.40 6-10	4.56 .91 1-6	1.5 .91 0-3	--

### 2.2 Reference standard

#### 2.2.1 Autism diagnostic observation schedule—second edition

The ADOS-2 assesses and diagnoses autism across a spectrum of age, developmental level, and language skills (3). In this study, Module 2 was administered by a trained professional with seven years’ experience of conducting ADOS-2 in schools and private practice, and with formal ADOS-2 training for clinical purposes. She conducted ADOS-2 under the supervision of another trained professional who had completed ADOS-2 advanced research training. The overall calibrated severity score (ADOS-CSS) of each child was reported based on the revised algorithms created by Hus et al. (28) for analyses. In comparison to the raw scores, the calibrated severity scores were less influenced by child characteristics (29), hence increasing their utility as indicators of social communication and repetitive behavior severity (28).

### 2.3 Stimuli and procedures

**Figure 1:**
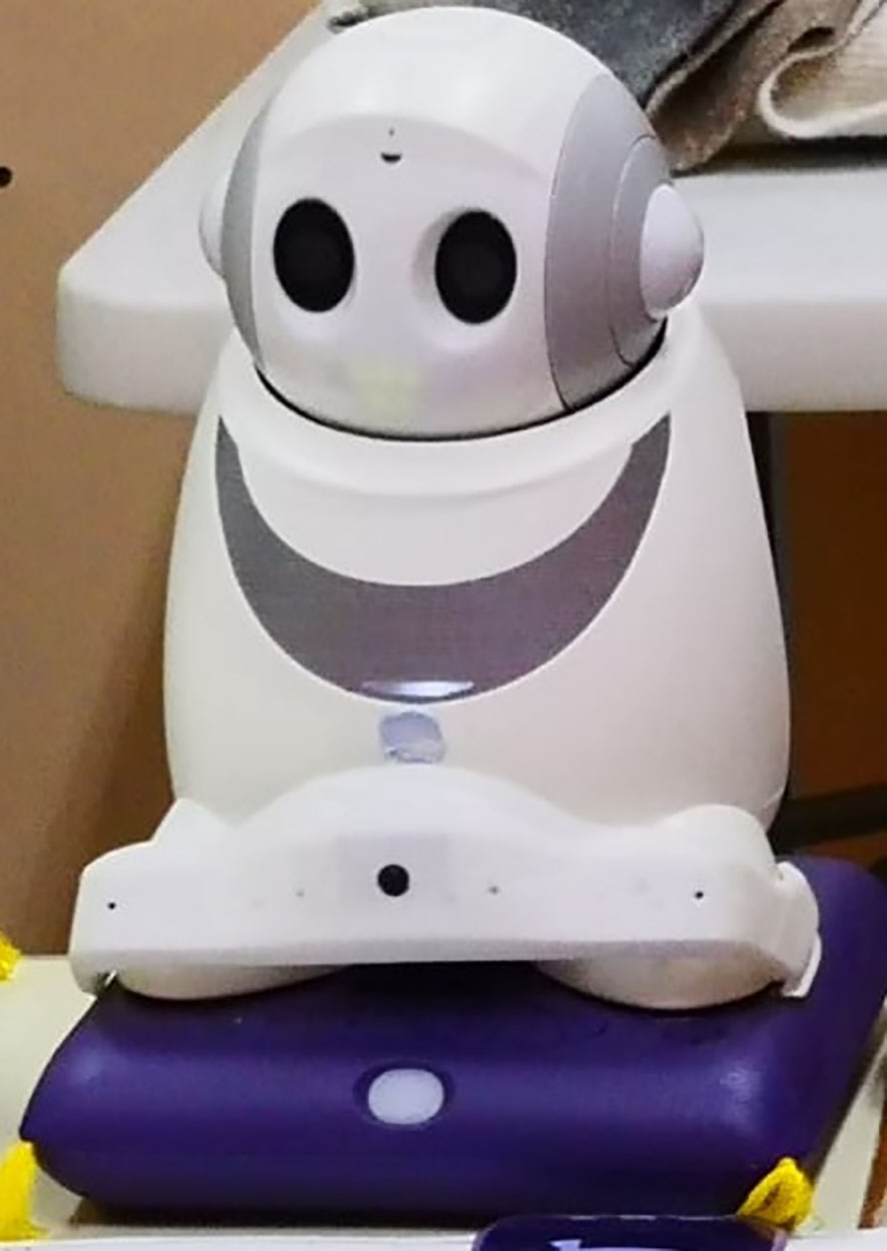
The social robot, HUMANE. A camera was installed on HUMANE to take frames of the child’s image and motor movements when it was talking to the child.

Same as So et al (11), HUMANE was programmed to detect the eye gaze toward the robot and repetitive motor manners. HUMANE, standing at a height of 25 cm and weighing 3.2 kg, possesses human-like attributes, including a baby-face appearance and voice vocalizations (see **Figure 1**). It has previously been employed in both autism and eldercare contexts (30, 31). Using the text-to-speech program, Murf AI, all six written story scripts were subsequently vocalized in Cantonese. Their sound clips were then uploaded to HUMANE.

HUMANE was installed with facial and movement recognition technologies developed by the IT corporation NEC (an international technology company) in the early 1990s. Its face recognition engine, empowered by NeoFace^®^ face recognition technology, can autonomously detect the focus of a child’s gaze through the camera installed on the robot. Detection is based on a unique algorithm that combines feature extraction, pattern recognition, and matching techniques to accurately identify and verify individuals. The NeoFace^®^ technology is used in a wide range of applications, including law enforcement, immigration control, access control, and retail activities in both indoor and outdoor settings. In 2021 NEC ranked first in the world in the most recent face recognition technology benchmarking test (FRVT Ongoing, *1) conducted by the U.S. National Institute of Standards and Technology (NIST). The system was evaluated with an accuracy rate of 99.78% for still images among 12 million people (*2). In NIST benchmark testing, NEC also ranked first following the face recognition benchmark in 2018 (FRVT2018, *3). Recently, NEC has also developed a posture engine that can autonomously detect the position of the child’s body based on another unique algorithm that extracts body landmarks.

HUMANE was programmed to narrate six stories, which were similar to those narrated in So et al (11) study, with each story composed of 15 to 20 sentences, and each clip lasting for six to seven seconds. The contents were similar across the six stories, which featured the daily life of Alan, a three-year-old boy. In one of the stories, Alan dined with his parents in a restaurant and learnt how to order food. HUMANE narrated three stories during the session of eye gaze detection and another three stories, with similar length and contents, during the session of repetitive motor manners detection. The order of eye gaze and repetitive movement detection programs was counterbalanced across participants.

The detection sessions were conducted in a treatment room in the affiliated university of the first author. The room was designed for the purposes of intervention and assessment of children. Prior to the start of each session in this study, the human experimenter would seat the child in a chair in front of HUMANE. The child was sitting 1.2 meters in front of HUMANE in the eye gaze detection program and 2 meters in front of HUMANE in the repetitive movement detection program. Before session began, the child was instructed to sit properly in front of HUMANE, with eyes looking at the robot and both hands resting at both sides of the body and both feet resting them on the floor. The experimenter was present together with the robot and the child, but she was standing at a distance from the child in order not to be captured by the robot.

Then the experimenter initiated the program, named the Autism Care Windows program, on a computer tablet. The program would then prompt HUMANE to begin the session, either the detection of atypical eye gaze or that of repetitive motor manners. After choosing the detection program, HUMANE operated autonomously without the involvement of the human experimenter. To begin the session, HUMANE commenced with a salutation and self-introduction, “Hi, (child’s name), I am Skype.” Then HUMANE would inquire whether the child would like to listen to a story narration by asking “Would you like to listen to a story?” Following confirmation, HUMANE would proceed to narrate the story while simultaneously monitoring the child’s gaze or repetitive motor manners.

During the eye gaze detection session, the camera captured images of each child participant during the robot’s narration and the program retrieved images from HUMANE at eight frames per second and continually relayed them back to NeoFace^®^, which was running in the cloud via a 5G mobile network. The technology processed the incoming frames, recorded the gaze results in a database for report generation, and sent metadata to HUMANE for vocalizing. Upon receiving the metadata, HUMANE decoded each message and analyzed what to do next. If the child discontinued eye contact with HUMANE for a predetermined interval of five seconds, HUMANE would stop the story narration and prompt the child with statements such as “Child, eyes on me, please!” or “Child, please look at me!” If the child re-established eye contact within one second of the prompt, HUMANE would provide positive feedback, with praises such as “Good looking! Well done!” or “You have done a good job! Thanks for looking at me!”. The off-focus timer was cleared, and HUMANE resumed narration. However, if the child failed to re-establish eye contact after the initial prompt, HUMANE would issue another prompt a second later. If this persisted for five minutes (for instance, if the child walked away or continued to gaze at something other than HUMANE), HUMANE would terminate the training session and bid the child farewell. On average, each session lasted for 10 minutes.

During the session of repetitive motor manners detection, the robot, equipped with a high-resolution camera and vision system, captured the child’s body position and assessed children’s body movements in real time. The detection program retrieved images from HUMANE at eight frames per second and continually relayed them back to the posture engine, which was running in the cloud via a 5G mobile network. The engine was trained to analyze hand mannerisms (e.g., hand flapping/waving) and complex mannerisms (e.g., body rocking, pacing back and forth, spinning circles). Then the engine sent metadata to HUMANE for vocalizing. HUMANE would prompt the child, “Child, please sit properly!”, when it detected improper postures, such as body rocking and spinning, for a predetermined interval of five seconds. If the child responded appropriately within a second, the robot provided positive feedback, “You have sat properly, well done!” and continued the narration. However, if the child did not correct their posture, the robot continued to prompt them.

**Figure 2:**
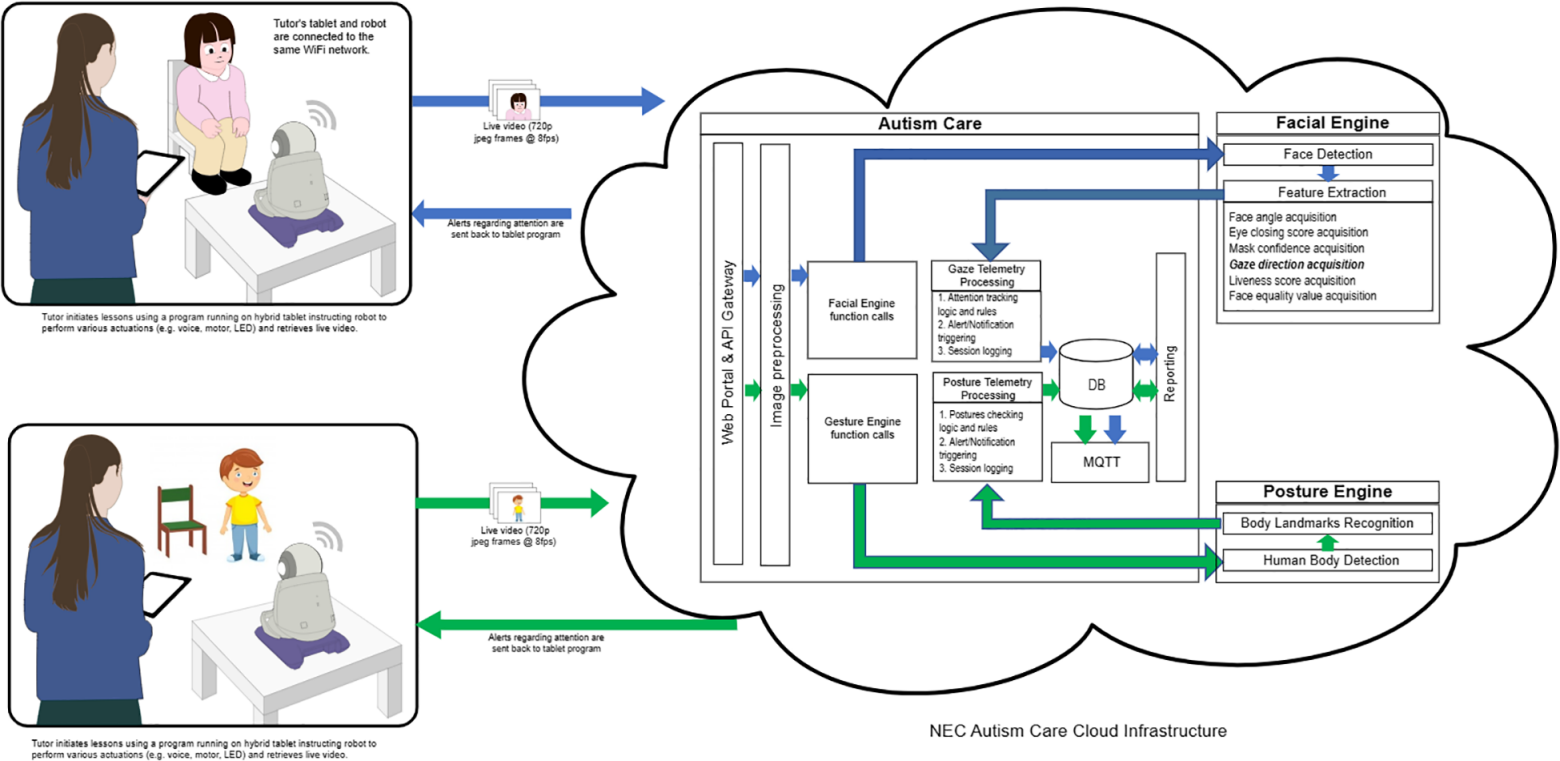
The mechanism shows how HUMANE autonomously detected the child’s eye gaze and motor movements and how it prompted and reinforced the child.

**Figure 2** depicts the procedures by which HUMANE autonomously detected the child’s eye gaze and repetitive motor movements, prompted the child, and reinforced the child in this study. All images were stored on the cloud server. Upon completion of each session, the Autism Care Program produced a record of the number of prompts and positive feedback that HUMANE had delivered to the child and the cumulative duration of their inattention (in seconds) during the session. Community members were not involved in the study.

### 2.4 Reliability

Previous research has shown the reliability of eye gaze detection across all pairs of human raters and HUMANE reached 0.90, indicating excellent interrater agreement (11). This study evaluated the reliability of the detection of repetitive motor movements by following the same procedures as So et al. (11). We recruited a group of four human raters, who were unaware of the research hypotheses. These raters were then trained by the first author to code the body posture of the child participants for each stored frame, with a total of 52,540 frames. The coding scheme was binary, with raters indicating whether the posture of the child was deviating from the starting position. Each time we picked a pair of coding completed by any of the two raters (e.g., Rater 1’s coding vs. Rater 2’s coding) on all stored frames and computed inter-rate reliability, named Cohen’s kappa coefficient (κ; 32). As there were four raters, there were six pairs of coding on all stored frames. The average Cohen’s κ across all pairs was.82 (SD = .06, ranging from.78 to.85). An average kappa score greater than 0.8 is usually considered to be sufficient to establish reliability for each pair of coding. We then measured the inter-rater reliability between the technology and each of the human raters, with the technology treated as an additional rater. We used the technology’s output as a threshold and then computed the inter-rater reliability between the technology and each of the human raters using Cohen’s κ. The average Cohen’s κ of all pairs between the technology and human raters was.85 (SD = .07; ranging from.82 to.88), usually considered to be almost perfect agreement. There was no significant difference between both sets of comparisons, with *t* (58) = 1.32, and *p* <.19.

### 2.5 Indexes and their discriminative power

The study aimed to validate the two individual indexes in each of the detection programs and composite indexes in both programs. For atypical eye gaze detection, the two individual indexes were the number of prompts made by the robot and cumulative duration of inattentiveness (in seconds). For repetitive motor movement detection, the two individual indexes were the number of prompts made by the robot and the cumulative duration of improper posture (in seconds). The two composite indexes were the robot prompts (i.e., the total number of prompts made by the robot in both detection programs) and duration (i.e., the total cumulative durations of inattentiveness and improper sitting posture). Analyses were conducted for both individual and composite indexes.

Separate Mann-Whitney U tests were conducted to examine whether children with ADOS-CSS scores below the cut-off point had a greater number of robot prompts and a longer cumulative duration at individual and composite levels than did the children with ADOS-CSS scores above the cut-off point. The cut-off point was pre-specified at four (29). In other words, those with ADOS-CSS scores of three or below are not likely to be on the autistic spectrum.

Additionally, we conducted separate diagnostic accuracy analyses, sensitivity and specificity tests for each of the individual and composite indexes. Their cut-offs, which intersected with the pre-specified cut-off of AQ-10-Child-HK (i.e., a score of 5), were derived from So et al (11) study. At the individual level, the cut-off for the number of prompts made by the robot in the atypical eye gaze detection was three and that for the cumulative duration of inattentiveness was 25 seconds. The cut-offs for the individual indexes in the detection of repetitive motor movements were the same. At the composite level, the cut-off for the number of robot prompts was six (the sum of the cut-offs for the number of prompts made by the robot in both detection programs) and the cut-off for the cumulative duration was 50 seconds (the sum of the cut-offs for the duration of inattentiveness and that of improper sitting posture).

For each index at the individual and composite level, sensitivity (its ability to detect autism when it is truly present) was calculated by the number of true positives divided by the total number of true positives and false positives. Similarly, for each index, specificity (its probability to exclude the disorder status in individuals who do not have autism) was calculated by the number of true negatives divided by the total number of true negatives and false positives. A goal of 80% sensitivity and specificity across autistic and non-autistic groups was set for each index, respectively. The Diagnostic Odds Ratios (DORs) for each of the indexes were also calculated (true positives x true negatives)/(false positives x false negatives). In respective indexes, we conducted the receiver operating characteristic (ROC) analysis to compute the Area under the Curve (AUC) to examine their validity or the discriminative power. A goal of AUC 0.7 to 0.8, considered acceptable, was set for each index at the individual and composite levels.

## 3 Results

**Figure 3:**
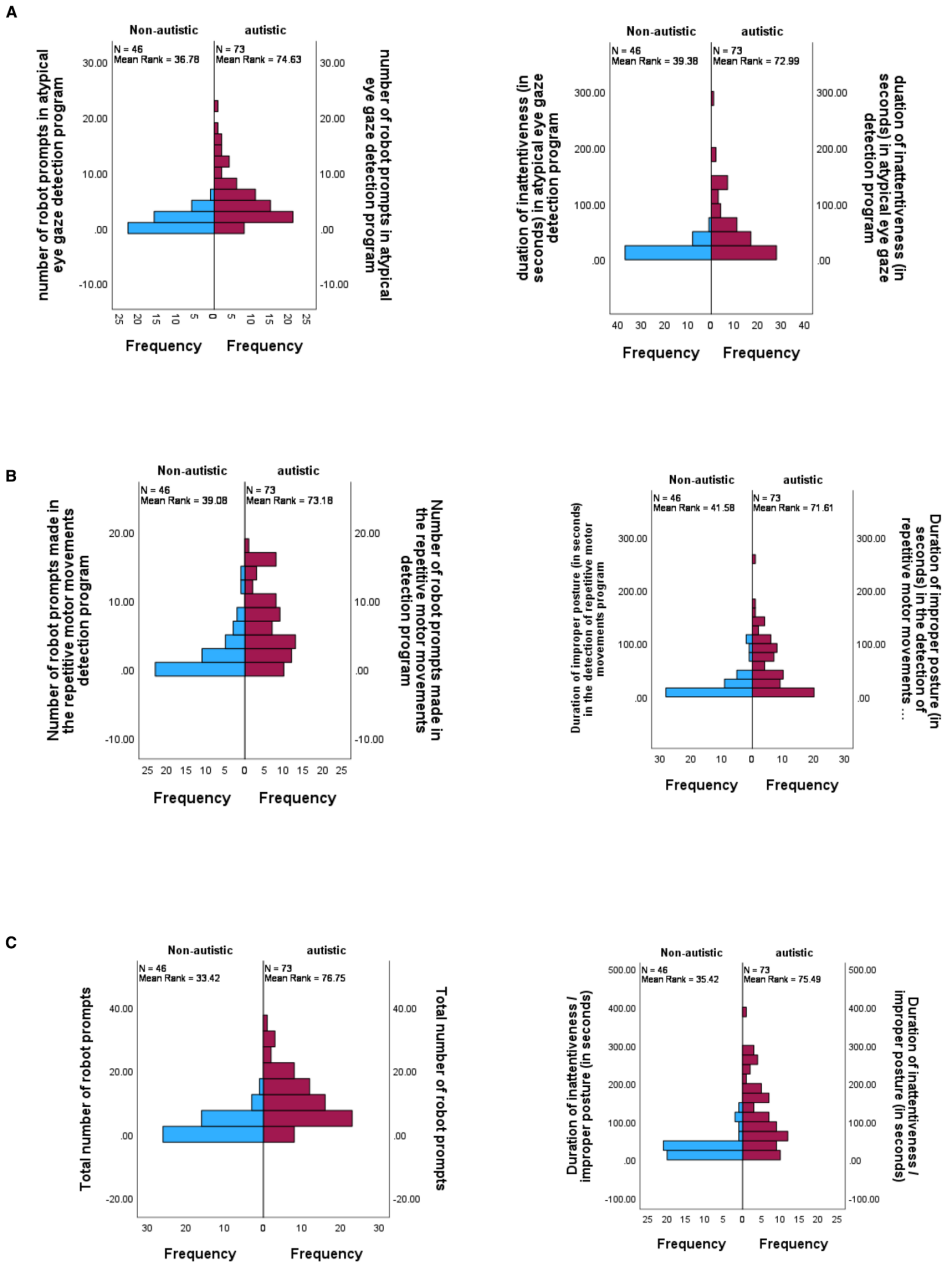
Mann-Whitney U test generated graphs used to visually inspect distributions of different indexes at the individual [**(A)** detection of atypical eye gaze; **(B)** detection of improper posture] and composite levels **(C)**. Frequency refers to the number of children obtaining a score in the corresponding number of prompts or duration of inattentiveness / improper posture in the individual and composite levels.

All children completed listening to the narrations by the robot and they did not show any problems interacting with the robot. **Table 2** shows the descriptive statistics for the severity of the children’s autism and the responses of the robot to the children’s eye gaze. Using a score of four as the cut-off point in the ADOS-CSS scores (29), 61.30% of the participating children scored on or above the cut-off point. Separate Mann-Whitney U tests were conducted to examine whether the participating children who scored on or above the ADOS-CSS cut-off point had more robot prompts and longer inattentive/improper posture duration than those below the cut-off point at both individual and composite levels. **Figure 3** presents the distribution for each of the indexes in the autistic and non-autistic groups. At the individual level, our results show that the number of prompts made by the robot in the atypical eye gaze detection program was significantly greater in the autistic group (mean rank = 74.63) than in the non-autistic group (mean rank = 36.78), U = 2747, z = 5.91, p <.001, r = .54. The duration of inattentiveness (in seconds) was significantly longer in the autistic group (mean rank = 72.99) than in the non-autistic group (mean rank = 39.38), U = 2627.50, z = 5.19, p <.001, r = .48. Regarding the detection of repetitive motor movements, the number of prompts made by the robot was significantly greater in the autistic group (mean rank = 73.18) than in the non-autistic group (mean rank = 39.08), U = 2641.50, z = 5.32, p <.001, r = .49. The duration of improper posture (in seconds) was significantly longer in the autistic group (mean rank = 71.61) than in the non-autistic group (mean rank = 41.58), U = 2526.50, z = 4.65, p <.001, r = .42. The effect sizes of the indexes at the individual level were medium. Similar patterns were found at the composite level. The total number of prompts made by the robot in both detection programs was significantly greater in the autistic group (mean rank = 76.75) than in the non-autistic group, (mean rank = 33.42), U = 2901.50, z = 6.69, p <.001, r = .61. The inattentive and improper posture duration detected by the robot was significantly longer in the autistic group (mean rank = 75.49) than in the non-autistic group, (mean rank = 35.42), U = 2809.50, z = 6.17, p <.001, r = .57. Both effect sizes were large.

**Table 2:** Means and SDs of autism assessment and various kinds of robot’s responses.

Variables	Mean	SD	Min	Max
ADOS- CSS	4.53	2.91	0	10
Detection of atypical eye gaze				
Number of prompts by HUMANE	3.32	4.12	0	21
Cumulative duration of inattention (in seconds)	38.52	48.61	0	286.60
Detection of repetitive motor movements				
Number of prompts by HUMANE	4.52	4.91	0	18
Cumulative duration of improper sitting posture (in seconds)	43.19	48.17	0	261.60
Composite number of prompts	7.82	7.57	0	34
Composite duration	81.58	80.07	0	384.90

We then calculated the sensitivity and specificity and Diagnostic Odds Ratios (DORs) of the indexes at the individual and composite levels (see **Table 3**). At the individual level, the number of prompts made by the robot in the atypical eye gaze and repetitive motor movements detection was test positive at three or above, and the cumulative inattentive/improper sitting posture was test positive at 25 seconds or above (11). The sensitivity of all four indexes at the individual level was low (.58,.62,.68, and.66), suggesting that the false negatives were high. Different results were found in specificity among all four indexes. The specificity of the indexes generated in the atypical eye gaze detection was high (.85 and.80) whereas that of the indexes in the detection of repetitive movements was low (.72 and.65). This suggests that false positives were higher in the detection of repetitive movements than in the atypical eye gaze detection. All DORs were greater than one, meaning that the autistic group had more robot prompts and a longer duration of inattentiveness/improper posture than the non-autistic group. DORs of the indexes generated in the atypical eye gaze detection program were higher than those of the indexes in the repetitive motor movements detection program.

**Table 3:** The decision matrix tables for the number of prompts made by the robot and the cumulative inattentive duration (in seconds) in the atypical eye gaze detection program (a); the number of prompts made by the robot and the cumulative duration of improper posture (in seconds) in the repetitive motor movements detection program (b); and the total number of prompts made by the robot and the cumulative inattentive/improper posture in both detection programs (c).

3a)		Number of prompts made by the robot		N
		Test outcome positive	Test outcome negative	
ADOS-CSS	Condition positive	True positive (43)	False negative (30)	73
	Condition negative	False positive (7)	True negative (39)	46
Sensitivity = .58	Specificity = .85	DOR = 7.99		

		Inattentive duration (in seconds)		N
		Test outcome positive	Test outcome negative	
ADOS-CSS	Condition positive	True positive (45)	False negative (28)	73
	Condition negative	False positive (9)	True negative (37)	46
Sensitivity = .62	Specificity = .80	DOR = 6.61		

3b)		Number of prompts made by the robot		N
		Test outcome positive	Test outcome negative	
ADOS-CSS	Condition positive	True positive (50)	False negative (23)	73
	Condition negative	False positive (13)	True negative (33)	46
Sensitivity = .68	Specificity = .72	DOR = 5.52		

		Improper posture duration (in seconds)		N
		Test outcome positive	Test outcome negative	
ADOS-CSS	Condition positive	True positive (48)	False negative (25)	73
	Condition negative	False positive (16)	True negative (30)	46
Sensitivity = .66	Specificity = .65	DOR = 3.6		

3c)		Total number of prompts made by the robot		N
		Test outcome positive	Test outcome negative	
ADOS-CSS	Condition positive	True positive (59)	False negative (14)	73
	Condition negative	False positive (5)	True negative (41)	46
Sensitivity = .81	Specificity = .89	DOR = 34.56		

		Total Inattentive/improper posture duration (in seconds)		N
		Test outcome positive	Test outcome negative	
ADOS-CSS	Condition positive	True positive (57)	False negative (16)	73
	Condition negative	False positive (5)	True negative (41)	46
Sensitivity = .78	Specificity = .89	DOR = 29.21		

Sensitivity and specificity and DORs of indexes at the composite level performed better than those at the individual level. At the composite level, the number of robot prompts was test positive at six or above, and the cumulative inattentiveness and improper sitting posture was test positive at 50 seconds or above. The sensitivity of the number of robot prompts and duration was close to.80 while the specificity of these two indexes was.89, both of which were greater than those at the individual level. The DORs of the indexes were at least 29, also higher than those at the individual level.

**Figure 4:**
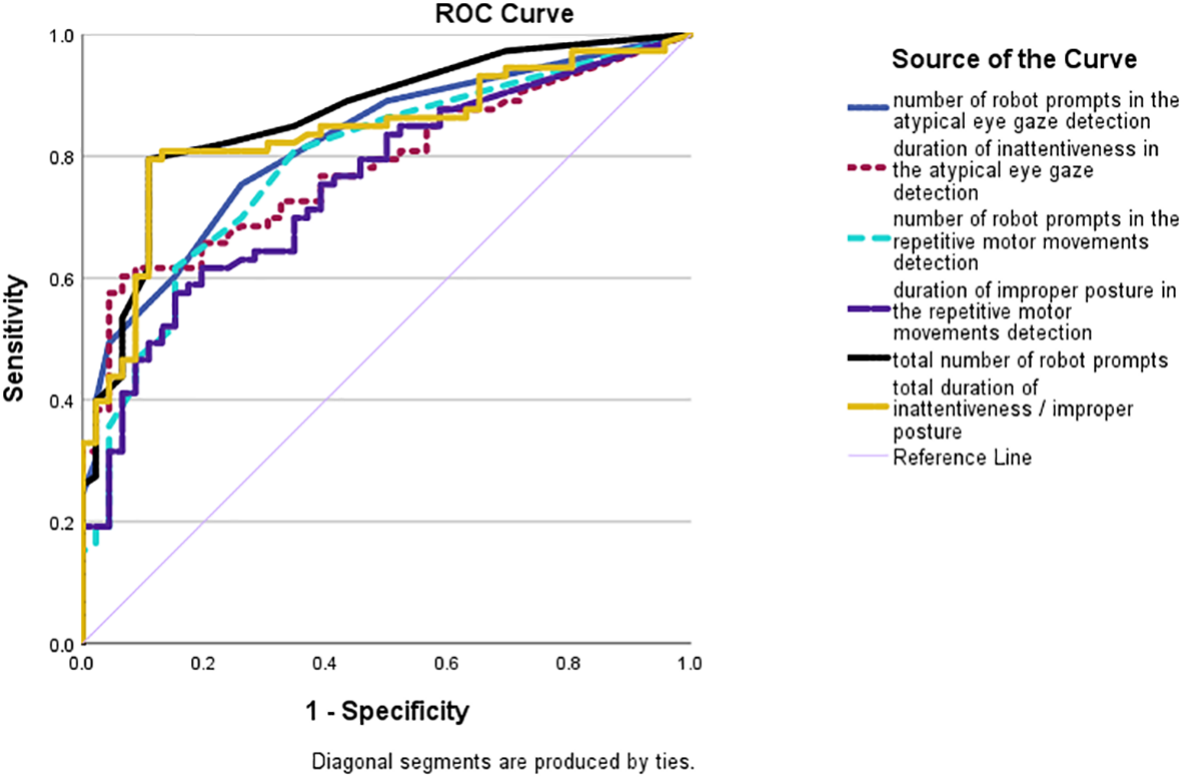
ROC curves of the number of prompts made by the robot in the atypical eye gaze detection (blue solid line); the cumulative duration of inattentiveness (dotted purple line); the number of robot prompts in the repetitive motor movements detection (dotted light blue line); the cumulative duration of improper posture (solid purple line); the total number of robot prompts in both detection systems (solid black line); and the total cumulative duration of inattentiveness and improper posture (solid yellow line).

Similar results were found in the ROC curve analyses (see **Figure 4**). The total number of robot prompts and the total inattentiveness and improper posture duration were competent in differentiating between the autistic and non-autistic groups with large AUCs of 0.86 (SE = .03; p <.001; 95% CI:.80-.93) and.84 (SE = .04, p <.001; 95% CI:.76-.91). The AUCs of individual indexes were lower, yet still higher than 0.7. Number of robot prompts in the atypical eye gaze detection: AUC = .82; SE = .04; p <.001; 95% CI:.74-.89; inattentive duration in the atypical eye gaze detection: AUC = .78; SE = .04; p <.001; 95% CI:.70-.86; number of robot prompts in the repetitive motor movements detection: AUC = .79; SE = .04; p <.001; 95% CI:.70-.87; improper posture duration in the repetitive motor movements detection: AUC = .75; SE = .04; p <.001; 95% CI:.67-.84.

To summarize, the indexes at the composite level generated by our robotic detection technology – the total number of robot prompts and cumulative inattentiveness and improper posture duration (in seconds) – could identify the presence or absence of autism. These indexes had a greater discriminative power than those at the individual level.

## 4 Discussion

This study aimed to develop and validate robotic technology for assessing autism. It was built upon previous research that developed a screening tool for autism using a social robot (11). Using standard diagnostic tool, ADOS-2, as a reference standard and the index tests previously established, our findings show that the total number of robot prompts and cumulative duration of inattentiveness/improper posture generated in the detection of atypical eye gaze and improper posture systems could identify the presence or absence of autism in 119 children aged three to six, with sensitivity and specificity of at least.80 and AUCs of at least.84. AUC above 0.8 is considered good for discrimination (33).

In So et al (11) study the robot HUMANE, installed with computer vision and linked with recognition technology, autonomously detected atypical eye gaze in children aged three to eight, which is one of the social communication and interaction impairments among autistic children. When the child looked away from HUMANE for a period, HUMANE prompted the child and praised them if they re-established eye gaze quickly after a prompt. The indexes of the number of robot prompts and duration of inattentiveness (in seconds) were tested against the reference standard, which was the score of a parental self-report, the Autism Spectrum Quotient. The sensitivity and specificity were high, showing that HUMANE may be able to screen for autism in the future.

This study has advanced the robotic screening tool developed in So et al.’s study in various ways. First, HUMANE was programmed to autonomously detect an additional autism feature of repetitive motor movements, a type of restrictive and repetitive behavior. In addition to the detection of atypical eye gaze, this study has autonomously captured autism features in the categories of social communication deficits and interaction as well as restricted and repetitive behavior listed in DSM-5 (2). The accuracy of detecting repetitive motor movement reached an excellent interrater agreement (>.80) between each pair of human raters and between the human rater and the technology. As an additional autism feature was detected, the present study was able to generate indexes at the composite level, which were the total number of prompts made by HUMANE and the cumulative duration of inattentiveness and improper posture.

The second advancement lies in the evaluation of the robotic detection technology for autism by testing the indexes at the composite level against the calibrated severity score of ADOS-2, one of the standard diagnostic tools for autism, in 119 children aged three to six. Two composite indexes – the number of prompts made by the robot and the cumulative inattentiveness and improper posture duration – were generated from the two detection programs. Our results show that the detection technology can identify the presence or absence of autism, with the average sensitivity close to 0.8, specificity reaching 0.89, and the average AUC reaching 0.85. This supports our first hypothesis. The sensitivity and specificity of composite indexes were higher than those of the individual indexes generated from atypical eye gaze or repetitive motor movement detection, indicating the composite indexes might have a greater discriminative power than indexes generated at the individual level. This supports our second hypothesis.

To meet the diagnostic criteria for autism according to DSM-5, a child must have persistent deficits in social communication and interaction as well as producing restricted and repetitive behavior. Therefore, assessing autism based on a single deficit (e.g., atypical eye gaze) limits the clinical applicability (18). This study showed that the discriminative power of indexes generated from one detection system is low. Our findings show the sensitivity of the number of robot prompts in identifying the presence or absence of autism based on the detection of one feature to be on average only.63, indicating high false negatives. Similar results were reported in the average sensitivity of the duration of inattentiveness/improper posture (.64). Merely looking at the number of prompts or duration of either atypical eye gaze detection or repetitive motor movements detection would miss a great number of children who were indeed diagnosed as being on the autism spectrum. Even more problematic, both detection systems made different decisions on autism assessment. For example, among the 119 participating children, only 72 of them received the same decision on autism assessment from both detection systems. There were 30 children who were considered non-autistic in the atypical eye gaze detection but were considered autistic in the repetitive motor movements detection. This might be attributable to the fact that children are easily engaged with social robots (34) and social robots may look less threatening and stimulating than a human (35), giving children less difficulty in establishing eye contact with the robot. However, these children might still display repetitive motor movements during the sessions, triggering HUMANE to prompt them. As a result, it is necessary to include the indexes generated from both detection systems. Our findings show that the average sensitivity across the two detection systems increased to.81.

In addition to advancing and validating the robotic detection technology for assessing autism, this study also overcomes the limitations of previous studies, including overreliance on humans in assessment and coding of children’s behaviors and the use of small sample sizes (e.g., 17, 22). The WoZ approach, where the human experimenter instructs the robot what to say and how to behave, is largely prevalent in the literature (e.g., 17, 18); however, autonomy is necessary and required when moving robotic technology forward (16). Social robots that operate autonomously might provide assessment and therapy at home or in school settings where neither researchers nor physicians are likely to be present, if validated by the proper institutions. In this study HUMANE was programmed to autonomously detect atypical eye gaze and repetitive motor movements and compute the cumulative duration with minimal involvement of humans administering the assessment. The detection data was coded and analyzed according to the algorithms. Additionally, HUMANE was programmed to interact with the child through giving prompts and praising them autonomously. The only duty assigned to the human experimenter was to start the Autism Care Windows program, while the detection, analyses, and interaction were accomplished by robotic technology.

The current robotic detection technology was evaluated in 119 children, including those diagnosed and not diagnosed to be on the autism spectrum and those suspected of having autism. To date, such a sample size is the largest among previous studies that have used a standard diagnostic tool as the reference standard. Given the heterogeneity of autism, future studies should enlarge the sample size and conduct repeated assessment on this group of samples in order to ensure the stability of the assessment.

Despite our promising findings, this study had a few limitations. First, the study devised a robotic technology that assesses autism by detecting atypical eye gaze and repetitive motor movements. Inclusion of various other autism features could provide a more accurate and comprehensive assessment of autism. We are in the process of programming an autonomous social robot to interact with the child while analyzing stereotypical speech behavior. Second, the procedure in which HUMANE narrated stories to children may be difficult to apply to children below the age of three who find it challenging to listen to stories. Different protocols need to be developed to cater for younger children. Third, during the session of detecting repetitive motor manners, the child was sitting 2 meters away from HUMANE, which was much further than the personal space intended for effective communication. Such long distance might influence the child’s attention to HUMANE. Future technological advancement is needed to shorten the distance between the child and HUMANE while ensuring the robot could detect the child’s body posture.

## 5 Conclusion

This study developed and evaluated robotic technology for assessing autism focusing on the autonomous detection of two features, atypical eye gaze and repetitive motor movements. The sensitivity and specificity of the indexes generated from such technology were tested against the standard diagnostic instrument in a large sample size and the findings show the discriminative power to be high.

## Data Availability

The raw data supporting the conclusions of this article will be made available by the authors upon reasonable request. It includes sensitive information such as autism diagnosis. Our participants involved vulnerable subjects.
